# GliSODin® prevents airway inflammation by inhibiting T-cell differentiation and activation in a mouse model of asthma

**DOI:** 10.3389/falgy.2023.1199355

**Published:** 2023-06-06

**Authors:** Martin Klein, Eleonore Dijoux, Marie-Aude Cheminant, Laurent Intes, Grégory Bouchaud

**Affiliations:** ^1^ Institut Universitaire de Cardiologie et de Pneumologie de Québec, Département de Médecine, Université Laval, Québec, QC, Canada; ^2^ Université de Nantes, CNRS, INSERM, l’institut du thorax, Nantes, France; ^3^ ISOCELL Nutra, Paris, France; ^4^ INRAE, Biopolymères Intéractions Assemblages (BIA), Nantes, France

**Keywords:** asthma, immunology, mouse model, dendritic cell, antioxidant

## Abstract

**Background:**

Asthma is a chronic inflammatory airway disease characterized by a prevailing type 2 inflammation, airway hyperresponsiveness, and mucus hypersecretion and is driven by various factors among which oxidative molecules, called reactive oxygen species (ROS), play a major role. Superoxide dismutases (SODs) are enzymes that constitute the first line of defense against ROS. Melon SOD-gliadin, which is known as GliSODin®, is commonly used as a nutritional supplement that has proven antioxidant properties.

**Objectives:**

In this study, we evaluated the efficacy and mechanism of action GliSODin® in the treatment of allergic asthma.

**Methods:**

House dust mite (HDM)-induced asthmatic mice were orally exposed to GliSODin®, and airway hyperresponsiveness, lung inflammation, *in vitro* T-cell polarization, *in vivo* T-cell reactivation, and blood immunoglobulin were investigated.

**Results:**

GliSODin® reduced airway hyperresponsiveness, lung innate and adaptive immune response, and HDM-specific IgE production. Coculturing CD4+ T-cell with HDM-sensitized dendritic cells and GliSODin® reduced T-cell polarization into Th2 and Th17 cells. Moreover, adoptively transferred CD4+ T cells from asthmatic mice exhibited a reduced reactivation of Th2 and Th17 cells following stimulation with HDM plus GliSODin®.

**Conclusion:**

GliSODin® abrogates asthma features and reduces CD4+ T-cell polarization and reactivation. Taken together, these data suggest that GliSODin® could be used for the management of asthma symptoms.

## Introduction

Antioxidants quench reactive oxygen species (1); however, their levels decrease with age, causing a corresponding increase in oxidative stress (2–4). Oxidation leads to the production of free radicals resulting in a chain reaction that causes damage to living organisms. Therefore, it is becoming progressively more important to consume antioxidants with food or as dietary supplements. In recent years, antioxidants have been referred to as a seventh category of nutrients, in addition to proteins, carbohydrates, lipids, vitamins, minerals, and dietary fiber. The accumulation of oxidative stress is thought to contribute to the development of obesity and other lifestyle-related diseases (5). Asthma, whose prevalence has constantly increased over the last few decades, has become one of the most prevalent airway diseases affecting more than 300 million people worldwide. Despite the efficacy of treatments, asthma still results in altered quality of life, morbidity, and economic burdens (6). Severe asthma represents 5%–10% of cases and results in permanent respiratory limitation, frequent exacerbations, and sometimes death (7). Asthma is characterized by increased reactive oxygen species (ROS) production, exceeding the protective capacity of the natural antioxidant systems, and inducing oxidative stress (8). Oxidative stress is caused by oxidative reactions that affect cells containing high levels of ROS (9). Since oxidative stress contributes to asthma severity (10), factors that reduce its severity should help prevent asthma. ROS are typically detoxified by antioxidant mechanisms such as those involving the enzymes superoxide dismutase (SOD), catalase (CAT), and glutathione peroxidase (GPx). SODs catalyze the dismutation of superoxide anion (O2^−^), a free radical involved in several reactions that produce a variety of ROS and reactive nitrogen species (RNS) from which many other secondary radical species can be generated (11). Melon SOD-gliadin (GliSODin®) is a patented oral formulation that combines a vegetarian source of SOD with a vegetable prolamin (gliadin) that not only protects SOD from gastric digestion, but also promotes the delivery of SOD to the mucosa of the small intestine (12). Several studies using melon GliSODin® have been conducted in several animal models and in human clinical trials. In animal studies, melon GliSODin® increased the activity of antioxidant enzymes in circulating blood (13) and decreased the production of inflammatory cytokines (14). In parallel, investigations that measure SOD level in asthmatic mice show a decrease in SOD activity in mouse model of asthma (15, 16) associated with a decrease in catalase and glutathione peroxidase (17). To the opposite, it has been demonstrated that GliSODin is able to increase SOD expression and activity (13, 14). Finally, mice that were orally fed with anti-oxidative products exhibited a decrease in lung inflammation and lesions in mouse model of asthma (18, 19). A study of children with asthma and dust mite allergy in Indonesia showed significant improvements in asthma-related symptoms when immunotherapy was combined with GliSODin®. Therefore, based on this information, we hypothesized that the immunomodulatory properties of melon GliSODin® could prevent and reduce oxidative stress, and that it can be used as a therapeutic agent to treat asthma. Here, we investigated the effect melon GliSODin® in a house dust mite (HDM)-induced asthma model in mice and their effects on the immune reaction during asthma.

## Material and methods

### Animal model

Female BALB/c By J mice were obtained from Charles River (France). The protocol was approved by the Ethics Committee on Animals Experimentation Pays de Loire (accreditation number 3455). The mice were housed in ventilated cages in the IRS-1 Experimental Therapeutics Unit with free access to water and a standard diet (SAFE A04) and a dark/light cycle of 12:12 h. The total extract of HDMs was obtained from Stallergenes Greer (Antony, France).

### Acute respiratory allergy to HDM

**Figure 1:**
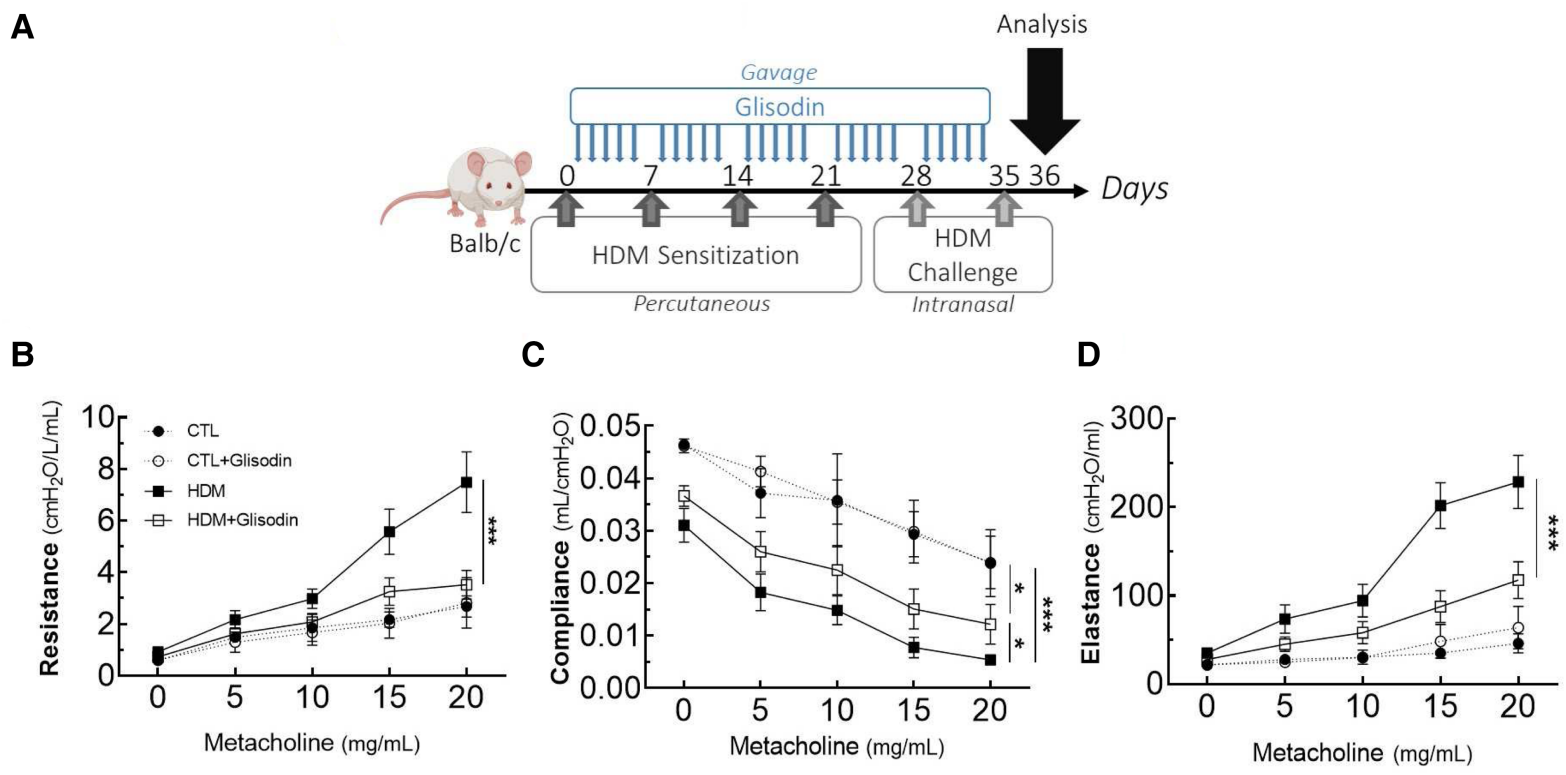
GliSODin® improves respiratory function in HDM-induced allergic mice via an anti-inflammatory effect. The mice were percutaneously sensitized to HDM to trigger an allergic reaction and received GliSODin® treatment (**A**). Airway resistance (**B**), compliance (**C**), and elastance (**D**) were measured by the forced oscillation technique. The black circle indicates the control (CTL), the white circle indicates the control plus GliSODin® treatment (CTL + Glisodin), the black square indicates HDM-sensitized mice (HDM), and the white square indicates HDM-sensitized mice treated with GliSODin® (HDM + Glisodin). The data are represented as the mean ± SEM, *n* = 6 mice per group for all groups, *p < 0.05, ***p < 0.005. HDM, house dust mites; GliSODin®, Melon SOD-gliadin.

Female BALB/c By J mice (aged 7 weeks) were sensitized once per week for 4 weeks with 500 µg HDM diluted in 20 µl of 70% DMSO/PBS (phosphate-buffered saline) by the percutaneous route (Figure 1A). After the sensitization phase, the mice were intranasally challenged (250 µg of HDM diluted with 40 µl of PBS) once per week for 2 weeks. Control mice received PBS only. The final analysis was performed 24 h after the last challenge (Day 36). GliSODin® was orally administered (gavage, 200 µl of GliSODin® diluted in water) daily 24 h after the first sensitization and until the end of the protocol (Day 36). This is equivalent to a dose of 5 mg per day of GliSODin®. Melon GliSODin® was used in the experiment; however, similar results were obtained using wheat GliSODin® (not shown).

### Airway hyperresponsiveness measurement

The day after the second challenge, invasive evaluations of airway resistance, compliance, and elastance were performed using the force oscillation technique (EMKA Technologies). The mice were anesthetized and intubated, and measurements were performed before and after the nebulization of increasing concentrations of methacholine (0–20 mg/ml).

### Bronchoalveolar lavage

Bronchoalveolar lavage (BAL) fluid was obtained by filling the airways with 1 ml of PBS buffer. The BAL fluid was centrifuged, and the pelleted cells were counted and analyzed by flow cytometry. The cytokine quantification of the BAL fluid was performed with Bio-plex Pro™ Mouse Cytokine Grp1 Panel 23-Plex (Bio-Rad®) according to the manufacturer's instructions using the Bio-plex® 200 system by Bio-Rad®. The BAL fluid chemokines were quantified using RANTES, eotaxin, KC, MIP1α, TARC, and MCP-1 ELISA kit (Bio-Rad Laboratories, Munich, Germany) according to the manufacturer's instructions.

### *In vitro* lymphocyte activation

After the “acute respiratory allergy to HDM” protocol, dendritic cells (DCs) were isolated from the bone marrow of BALB/c (BMDCs) and cultured *in vitro* with GliSODin® (5 mg/ml) in BMDC medium (RPMI1640, 1% penicillin–streptomycin, 1% L-glutamine, 1% sodium pyruvate, 1% HEPES, 1% MEM non-essential amino acid, B-mercapto-ethanol 100 µl/L of 100 nM, and SVF 10%) supplemented with 20 ng/ml GM-CSF (premium grade, Miltenyi Biotec). After 7 days of culture, HDM was added (10 µg/ml). After 8 days of culture, CD4+ and CD8+ (LT) lymphocytes were isolated from the spleens of mice after the “acute respiratory allergy to HDM” protocol and were enriched by negative selection with EasySep™ Mouse CD4+ and CD8+, T-Cell Isolation Kits (StemCell Technologies). The LT CD4+ or LT CD8+ were added to dendritic cells and cocultured. Two days later, the LT CD4+ and CD8+ differentiation profiles were analyzed by flow cytometry.

### Adoptive transfer

The LT CD4+ cells were isolated from the spleens of BALB/c expressing a congenic Ly5.1 marker mouse after the “acute respiratory allergy to HDM” protocol as described previously. 2–3.10^6^ LT CD4+ cells were stained with CFSE (Invitrogen) and intravenously injected into other WT BALB/c mice expressing the standard Ly5.2 marker. The following day, the mice were administered GliSODin® by gavage for 6 subsequent days. The mice were intranasally challenged (250 µg of HDM diluted with 40 µl of PBS) 3 and 6 days after adoptive transfer. The day after the last challenge, LT CD4+ expansion was analyzed by flow cytometry with congenic marker allowing the identification of donor cells.

### Flow cytometry

The lungs were removed and crushed with a Ten Broeck tissue grinder (Wheaton™) to obtain a single-cell suspension. The lung pellet was suspended in 1 ml of RBC lysis buffer (eBioscience™) for 8 min at room temperature and mixed with 9 ml of FACS buffer (DPBS without Ca^2+^ and Mg^2+^, 1% EDTA, 1% fetal bovine serum). The lung cell pellet was suspended in 600 µl of FACS buffer. The total cells were counted on KOVA slides. The BAL fluid cells were stained with the following surface markers: CD3-PerCP Cy5.5, CD4-APC-Cy7, CCR3-Alexa647, F4/80-FITC, Ly6G-BV421, Siglec F-BV510, and Ly6C-PE. T cells in the lung were stained with the following surface markers: CD3-PerCP Cy5.5, CD4-APC-Cy7, CD8-FITC, and CD25-BV510. The cells were fixed and permeabilized using a Cytofix/Cytoperm Kit (BD Biosciences) and stained with FoxP3-Alexa647, GATA3-PE-Cy7, and RORγT-PE. The LT CD4+ and CD8+ cells were stained with IFN-γ-FITC, Foxp3-PE, CD4-PerCP Cy5.5, CCR4-PE-Cy7, IL-4-Alexa647, CD8, APC-Cy7, IL-17-BV421, and CD25-BV510. For adoptive transfer, the LT CD4+ cells were stained with CFSE (FITC), Ly5.1-PE, CD4-PerCP Cy5.5, CCR6-PE-Cy7, RORγT-Alexa647, CD8-APC-Cy7, and IL-17RA-BV510. The cells were identified by flow cytometry on a Fortessa X20 cytometer (BD Biosciences). Data were acquired using DIVA software (BD Biosciences) and analyzed with FlowJo (TreeStar, V10.5.3). The gating strategies are shown in Supplementary Figure S3.

### Serum analysis

Blood was collected by cardiac puncture, and serum was isolated. The serum levels of total and specific Derf1 IgE were quantified by indirect ELISAs. A total of 96 well plates were coated with 2.5 µg/ml natural Derf1 (Indoor Biotechnology, NA-DF1-1) and incubated overnight at 4°C. Then, the wells were washed with PBS-0.1% Tween, saturated with PBS-0.1% Tween-0.5% gelatin (PBS-T-G) for 2 h at 37°C, washed, filled with serum diluted by 40x in PBS-T-G, and incubated overnight at 4°C. Specific Derf1 IgE was revealed with anti-mouse IgE (Cliniscience) coupled to AP. Substrate MUPs (Merck, Sigma-Aldrich) were added and incubated for 1 h and 30 min at room temperature in the dark, and fluorescence was measured at an excitation wavelength of 360 nm and an emission wavelength of 440 nm with a Varioskan LUX 3,020-956 (Thermo Fisher Scientific). The relative fluorescence was determined compared with that of control mice. MCP-1 levels were quantified using Bio-plex Pro™ Mouse Cytokine Grp1 Panel 23-Plex (Bio-Rad®) as BAL fluid analysis.

### Histology

Lungs were fixed in 4% paraformaldehyde in PBS for at least 48 h, embedded in paraffin, and cut and stained by the MicropiCell Platform (SFR Bonamy, IRS-UN, Nantes). Large lung scans were performed with a Nikon Eclipse Ti2 microscope (NIS-Element). The histological score was based on the following criteria: integrity and hyperplasia of the lung epithelium (measure of the increase in epithelial thickness), mucus production, lung inflammatory infiltrate (observation of inflammatory infiltrate or nodules), and bronchial smooth muscle remodeling (measure of the increase in smooth muscle thickness). Each of the four criteria was scored on 5 points with a relative comparison with control mice, for a final score of 20 points.

### Statistical analyses

The data were analyzed using GraphPad Prism 6.0 (La Jolla, CA, United States). Values are expressed as the mean ± SEM and were compared using non-parametric ANOVA with a Kruskal–Wallis test. A *p*-value of less than 0.05 was considered significant. A total of five to eight mice per group were used.

## Results

### GliSODin® prevents airway dysfunction and inflammation in asthma

**Figure 2:**
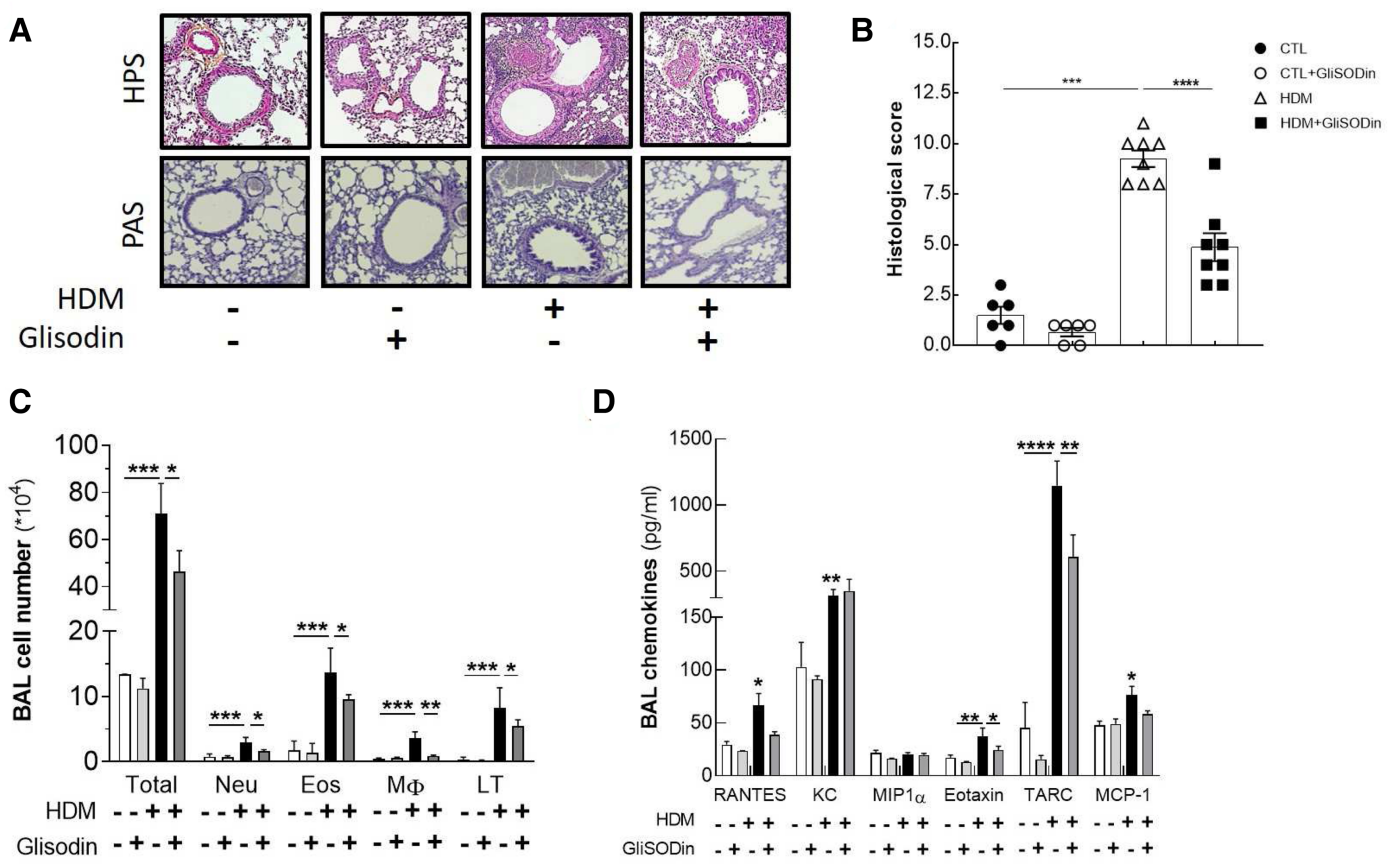
GliSODin® reduces lung remodeling and infiltration. Lung sections were stained with HPS after paraffin inclusion and were observed under a microscope (**A**). The histological score was calculated by measuring bronchial epithelial thickness, mucus production, inflammatory infiltration, and bronchial smooth muscle thickness (**B**). Lung infiltration was assessed by quantitative flow cytometry analysis of eosinophils (EO), neutrophils (Neu), macrophages (M), and CD4+ T lymphocytes (LT) present in the BAL fluid (**C**). The chemokine concentrations in the BAL fluid were quantified by ELISA kit (**D**). The white bar indicates the control (CTL), the light gray bar indicates the control plus GliSODin® treatment (CTL + Glisodin), the black bar indicates HDM-sensitized mice (HDM), and the gray bar indicates HDM-sensitized mice plus GliSODin® treatment (HDM + Glisodin). The data are represented as the mean ± SEM, *n* = 6 mice for CTL and CTL + Glisodin groups and eight mice for HDM and HDM + Glisodin groups, *p < 0.05, **p < 0.01, ***p < 0.005, ****p < 0.001. Comparison between groups are indicated by draw line or with all other groups when no line. HPS, hematoxylin-phloxine-saffron; GliSODin®, Melon SOD-gliadin; HDM, house dust mites; BAL, bronchoalveolar lavage.

**Figure 3:**
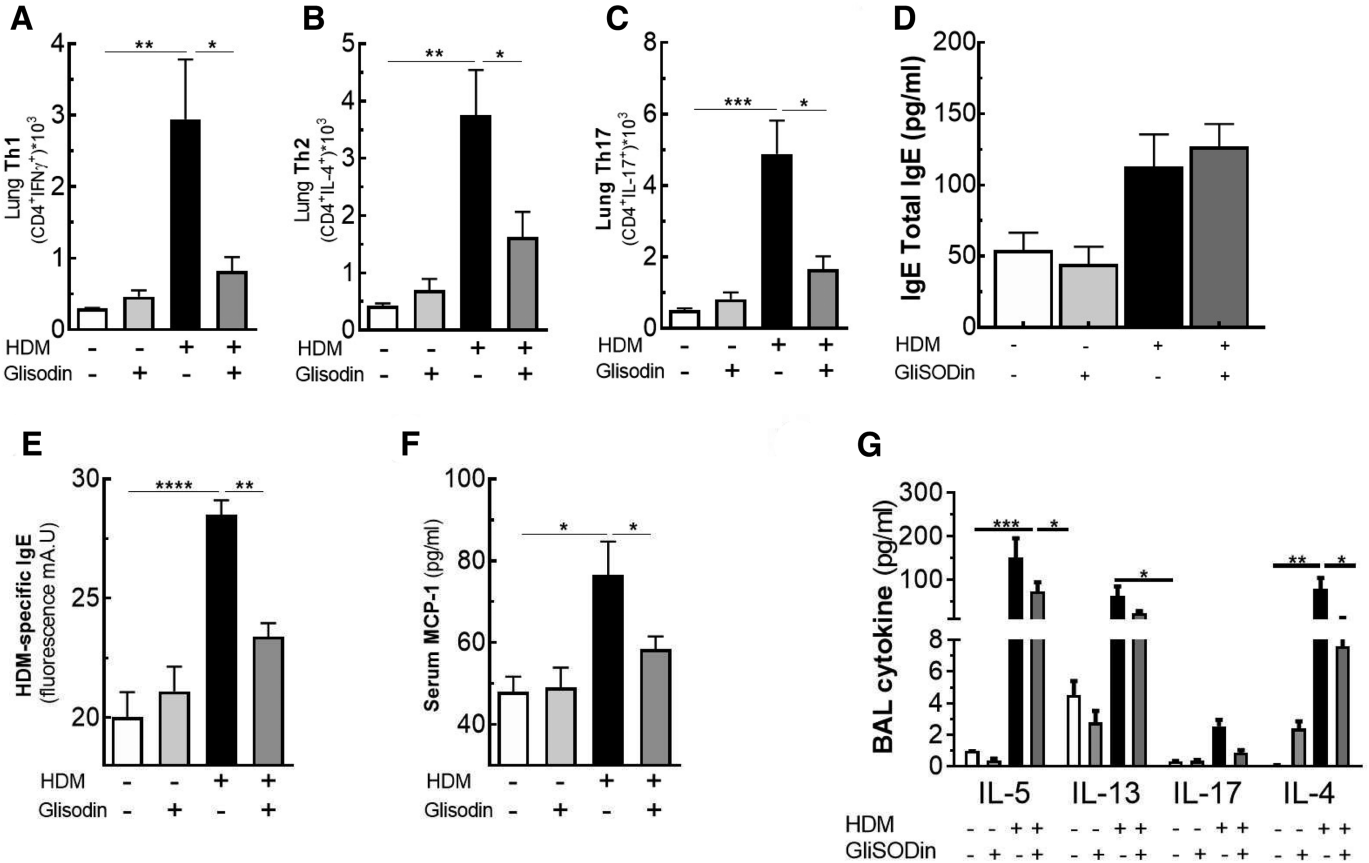
GliSODin® improves the anti-inflammatory effect on the T helper lymphocyte response. T helper 1 (Th1) lymphocytes (CD4+ IFNγ+) (**A**), T helper 2 (Th2) lymphocytes (CD4+ IL-13+) (**B**), and T helper 17 (Th17) lymphocytes (CD4+ IL-17+) in the lung were quantified by flow cytometry (**C**). The total and specific Derf1 IgE levels were quantified by ELISA (**D** and **E**). MCP-1 levels in serum (**F**) and the BAL fluid levels of the cytokine IL-5, IL-13, IL-17, and IL-4 were quantified by Bio-plex technology (**G**). The white bar indicates the control (CTL), the light gray bar indicates the control plus GliSODin® treatment (CTL + Glisodin), the black bar indicates HDM-sensitized mice (HDM), and the gray bar indicates HDM-sensitized mice plus GliSODin® treatment (HDM + Glisodin). The data are represented as the mean ± SEM, *n* = 6 mice per groups for all groups, *p < 0.05, **p < 0.01, ***p < 0.005, ****p < 0.001. GliSODin®, Melon SOD-gliadin; BAL, bronchoalveolar lavage; HDM, house dust mites.

To investigate the therapeutic potential of GliSODin®, we first measured its effects on a mouse model of HDM-induced airway inflammation (Figure 1). We used a previously described model harboring mixed Th2/Th17 inflammation associated with eosinophilic and neutrophilic infiltration (Figure 1A). Mice that received GliSODin® by oral gavage during the experimental protocol exhibited a decrease in lung resistance in response to methacholine compared with asthmatic mice and a similar level as control mice (Figure 1B). In parallel, mice receiving GliSODin® display an increase in compliance compared with asthmatic mice but remain significantly decreased compared with control mice (Figure 1C). Finally, lung elastance was significantly decreased in mice receiving GliSODin® compared with asthmatic mice to reach a level comparable with control mice (Figure 1D). Subsequently, pulmonary lesions were investigated (Figure 2). GliSODin®-treated asthmatic mice exhibited less perivascular and peribronchial cell infiltration, mucus production, and epithelial cell hyperplasia than asthmatic mice as shown by histological images (Figures 2A,B). Consistent with lung histological scoring, inflammatory cells in the BAL fluid of mice that received GliSODin® exhibited a decrease in macrophages, eosinophils, neutrophils, and lymphocytes (Figure 2C). This decrease in innate cells was associated with a reduced the BAL fluid level of the chemoattractant RANTES, eotaxin, TARC, and MCP-1 in asthmatic mice treated with GliSODin® compared with the untreated mice. However, the level of the chemoattractant molecule KC was still high even after the asthmatic mice were treated with GliSODin® (Figure 2D). We then measured inflammatory cells in the lung (Figure 3). As expected, asthmatic mice exhibited an increase in Th1, Th2, and Th17 cells compared with control mice. According to their inflammatory status, GliSODin®-treated mice showed a decrease in these three T helper (Th) lymphocytes populations compared with allergic mice and reached a level that was not significantly different from that of control mice (Figures 3A–C). Then, we explored the effect of GliSODin® on circulating total and HDM-specific IgE and MCP-1 levels, which reflects the Th2 humoral response and allergic markers (Figures 3D–F). Although asthmatic mice had a higher level of HDM-specific IgE than control mice, GliSODin®-treated mice exhibited a level of HDM-specific IgE that was similar to the control (Figure 3E). However, no significant effect of GliSODin® was observed on the total IgE level suggesting a multi modal effect (Figure 3D). In contrast, asthmatic mice had lower levels of HDM-specific IgA than control mice, and GliSODin®-treated mice exhibited levels of HDM-specific IgA that were similar to control (Supplementary Figure S1A). However, asthmatic and GliSODin®-treated mice exhibited the same levels of HDM-specific IgG1 and IgG2a, and IgG1 and IgG2a levels were higher and lower, respectively, than the control (Supplementary Figure S1B,C). Regarding MCP-1 levels, asthmatic mice had a higher level than control mice. Conversely, GliSODin®-treated mice had reduced levels of MCP-1 compared with asthmatic mice (Figure 3F). Our results demonstrate that GliSODin® treatment considerably reduced lung inflammation, airway hyperresponsiveness (AHR), and allergen-specific IgE, which are the three cardinal characteristics of allergic asthma. Finally, we confirmed our results by measuring Th-associated cytokines (Figure 3G). As expected, asthmatic mice exhibited increased levels of Th2- (IL-5, IL-13, and IL-4) and Th17 (IL-17)-associated cytokines compared with control mice. In contrast, GliSODin®-treated mice exhibited a less-pronounced increase in those cytokines. These promising results led us to investigate the mechanism of GliSODin®.

### GliSODin® inhibits Th2 and Th17 differentiation *in vitro*

**Figure 4:**
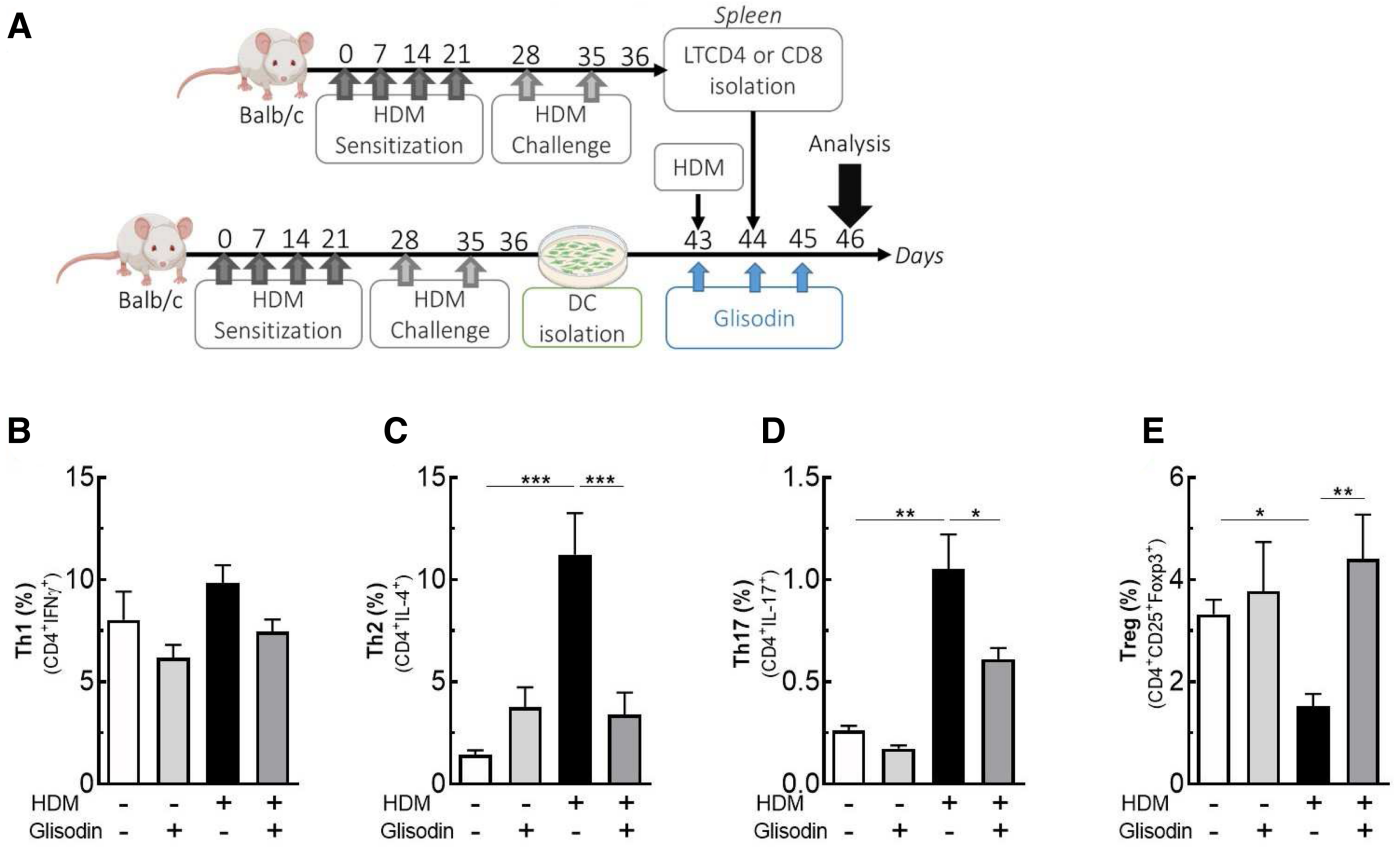
GliSODin® inhibits Th2 and Th17 differentiation *in vitro*. Dendritic cells were isolated from HDM-allergic mice, cocultured with LT CD4+, HDM, and GliSODin® (**A**). T helper 1 (Th1) lymphocytes (CD4+ IFNγ+) (**B**), T helper 2 (Th2) lymphocytes (CD4+ IL-4+) (**C**), T helper 17 (Th17) lymphocytes (CD4+ IL-17+) (**D**), and T regulatory (Treg) lymphocytes (CD4+ CD25+ Foxp3+) (**E**) in the lung were quantified by flow cytometry. The white bar indicates the control (CTL), the light gray bar indicates the control plus GliSODin® treatment (CTL + Glisodin), the black bar indicates HDM-sensitized mice (HDM), and the gray bar indicates HDM-sensitized mice with GliSODin® treatment (HDM + Glisodin). The data are represented as the mean ± SEM, *n* = 8 mice per group for all groups, *p < 0.05, **p < 0.01, ***p < 0.005, ****p < 0.001. HDM, house dust mites; GliSODin®, Melon SOD-gliadin.

To further elucidate how GliSODin® inhibits Th2 and Th17 responses in asthma, we analyzed *in vitro* its effect on T-cell differentiation (Figure 4A). We cocultured BMDCs and T cells from BALB/c mice in the presence of HDM extract and/or GliSODin®. When DCs were incubated in the presence of HDM extract, the frequency of CD4 + IFN-producing Th1 cells did not vary among experimental conditions (Figure 4B) nor did that of CD8+ lymphocyte (Supplementary Figure S2). However, CD4 + IL-4-producing Th2 cells increased compared with those of T cells cultured with control DCs (Figure 4C). In contrast, no increase in Th2 cells was observed when DCs were incubated with HDM extract and GliSODin®. Similar results were observed in CD4+IL-17A+ cells (Figure 4D). Interestingly, CD4+CD25+Foxp3+ Treg were decrease by HDM conditions but not in HDM + GliSODin® conditions (Figure 4E). These results suggest that GliSODin® is able to counteract the ability of HDM to induce Th2 and Th17 differentiation in T cells affecting on DC activation.

### GliSODin® inhibits T-cell reactivation *in vivo*

**Figure 5:**
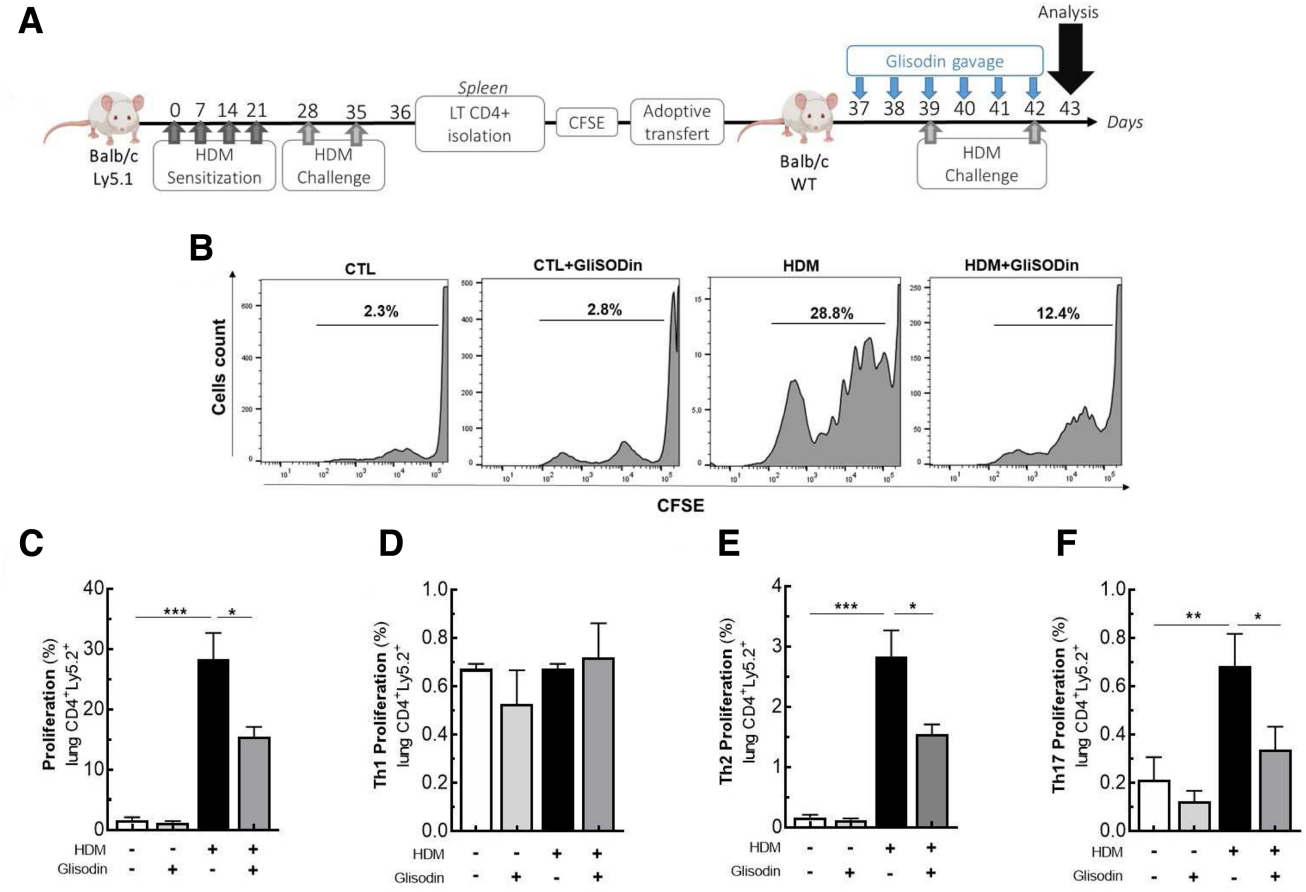
GliSODin® inhibits lymphocyte reactivation and proliferation during allergic reactions in mice. Adoptively cell transfer experiment was realized where LT CD4+ lymphocytes from HDM allergic mice (Ly5.1), stained with CFSE are IV transferred to another wild type Balb/c mice. The Balb/c mice were treated with GliSODin® treatment and two HDM challenges **(A)**. CD4+T lymphocyte proliferation was quantified by flow cytometry **(B)** and the Tcells **(C)** and its subpopulation Th1 **(D)**, Th2 **(E)** and Th17 **(F)** frequency was calculated. The white bar indicates the control (CTL), the light grey bar indicates the control plus GliSODin® treatment (CTL+Glisodin), the black bar indicates HDM sensitized mice (HDM), and the grey bar indicates HDM sensitized mice plus GliSODin® treatment (HDM+Glisodin). The data are represented as the mean ± SEM, n=5 mice per group for all groups, *p<0.05, **p<0.01, ***p<0.005, ****p<0.001.

Based on our previous results on mouse BMDCs and T-cell polarization, we investigated the effects of GliSODin® on T-cell reactivation (Figure 5). CD4+ cells from asthmatic mice were isolated, labeled with CFSE, and adoptively transferred into Ly5.1^+^ BALB/c mice. The following day, the mice were treated with or without GliSODin® by daily gavage until the day before analysis and were restimulated with HDM on days 3 and 6 after adoptive transfer (Figure 5A). Following HDM restimulation, CFSE labeling showed that lung Ly5.2^+^ CD4^+^ T-cell adoptively transferred from asthmatic mice proliferate more than Ly5.2^+^ CD4^+^ T cells from control mice. Interestingly, Ly5.2^+^ CD4^+^ T cells from asthmatic mice treated with GliSODin® and HDM extract proliferated less than those from HDM restimulated mice. Ly5.2^+^ CD4^+^ T-cell from control mice treated with GliSODin® alone exhibited a proliferation rate similar to that of cells from control mice (Figures 5B,C). To further investigate which CD4^+^ T-cell subpopulation was modulated, we analyzed the Ly5.2^+^ Th1, Th2, and Th17 T cells. We first observed that the Ly5.2^+^ CD4^+^ Th1 cells frequency was not altered by HDM restimulation or GliSODin® treatment in HDM, or HDM and GliSODin®-treated mice (Figure 5D). However, the Ly5.2^+^ CD4^+^ Th2 cells frequency in asthmatic mice was increased following HDM restimulation compared with control mice. By contrast, the Ly5.2^+^ CD4^+^ Th2 cells frequency in asthmatic mice was decreased after HDM restimulation and GliSODin® treatment compared with that in asthmatic mice treated with HDM restimulation alone (Figure 5E). We observed the same changes in Ly5.2^+^ CD4^+^ Th17 cells frequency in each group (Figure 5F). These results show that GliSODin® prevents Th2 and Th17 cells reactivation *in vivo* after allergen re-exposure.

## Discussion

**Figure 6:**
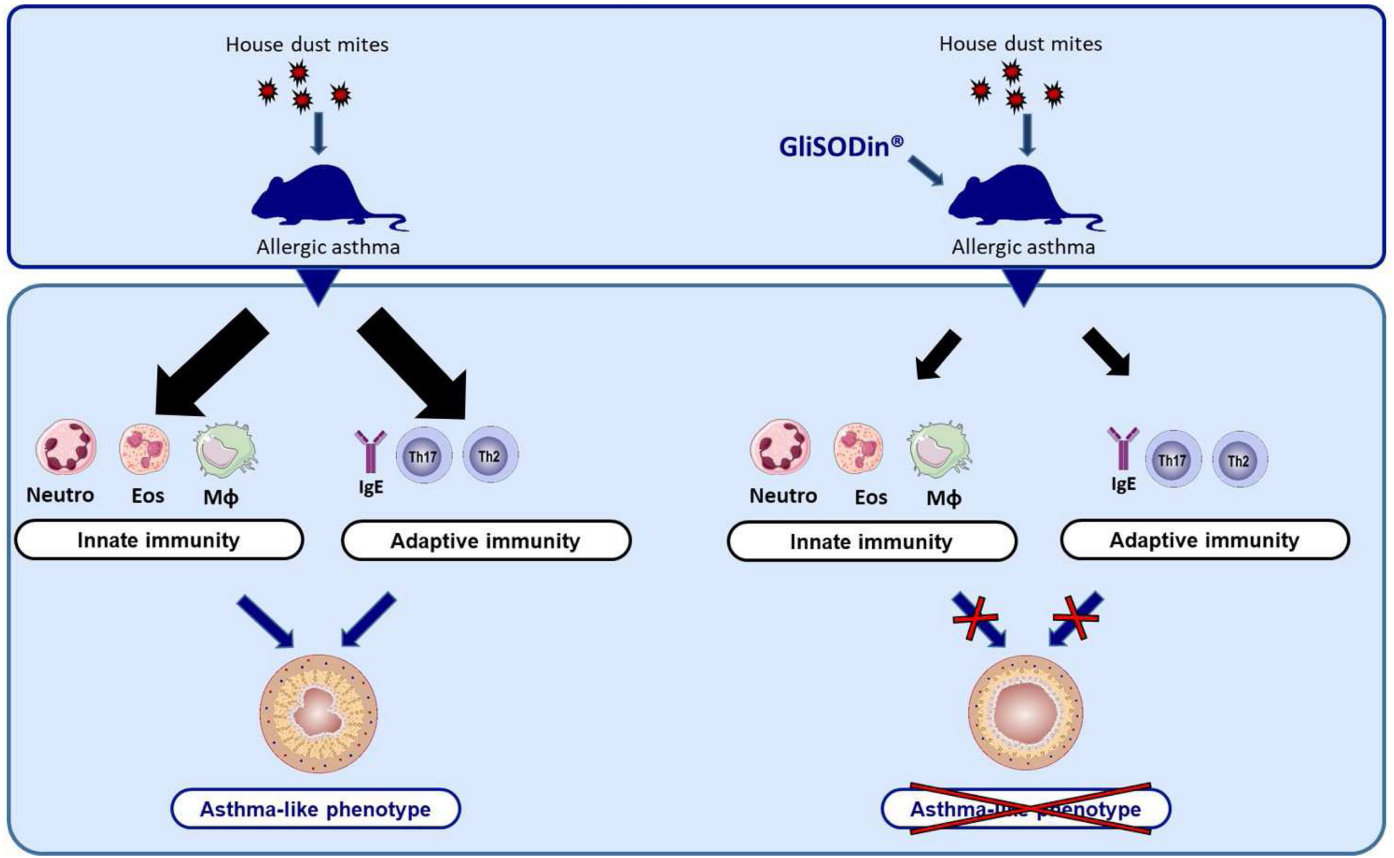
GliSODin® protects against allergic asthma in mice. After sensitization and challenge with house dust mites, the mice exhibited an inflammatory response characterized by innate immune cell infiltration and an adaptative immune response. In contrast, mice that were orally treated with GliSODin® exhibited reduced innate and adaptative immune responses resulting in a decrease in asthma-like symptoms. GliSODin®, Melon SOD-gliadin.

In the present study, we first demonstrated that GliSODin® supplementation prevents AHR, lung inflammatory infiltration, Th2 responses, and HDM-specific IgE production, which are cardinal features of allergic asthma. Moreover, we found that GliSODin® reduced pro-inflammatory chemokine and cytokine levels in the lung (Figure 6). Mechanistically, we showed that GliSODin® inhibited Th2 and Th17 cell polarization, proliferation, and reactivation. During asthma, ROS are responsible for epithelial damage and bronchial AHR (20). Here, we demonstrated for the first time that GliSODin® prevented HDM-induced structural alterations during asthma and AHR. Bronchial AHR is sustained by chemokines such as eotaxin, RANTES, and MCP-1, which promote recruitment and activation of eosinophils, mast cells, basophils, and macrophages in the lung (21). Eotaxin and MCP-1 can be induced by ROS (22, 23), and RANTES promotes ROS production by eosinophils (24). In the present study, we observed that there is no lung infiltration in HDM-induced asthmatic mice treated with GliSODin® at the microscopic level. Consistent with this finding, the BAL fluid analysis showed control-like levels of eotaxin, RANTES, MCP-1, eosinophils, neutrophils, and macrophages in asthmatic mice treated with GliSODin®, indicating the ability of GliSODin® to prevent the induction of chemokines and by extension the recruitment of innate cells that produce ROS and are responsible for AHR in the lung. Inflammation is another component of asthma pathophysiology and results from innate and adaptive inflammatory cells accumulation and activation in the lung. We demonstrated that in addition to preventing innate immune cells accumulation in the lung, GliSODin® prevented lung accumulation of adaptive inflammatory Th2 and Th17 cells. TARC is a chemokine ligand that attracts and binds to CCR4-expressing cells. Th2 and, to a lesser extent, Th17 cells express CCR4 (25) and can migrate to tissues in which TARC is expressed (26). In addition, TARC can be induced by ROS through NF-*κ*B signaling (27). In our study, the absence of Th2 and Th17 cell infiltration in the lung may be due to the lower levels of TARC in the BAL fluid of GliSODin®-treated asthmatic mice than the untreated asthmatic mice.

DCs are the bridge between innate and adaptive immunity, and these cells specialize in presenting antigen fragments to naïve T cells through major histocompatibility complexes I and II (MHCI and MHCII, respectively). DC activation is characterized by the expression of the costimulatory molecules CD80, CD86, CD40, and MHCII that enable DCs to prime naïve T cells and polarize them into specific Th cells according to the presented antigen (28). During asthma, DCs are able to polarize naïve T cells into Th2 and/or Th17 cells that sustain an inflammatory environment (29). Our *in vitro* results demonstrated that coculturing GliSODin®-primed DCs with naïve T cells prevented T-cell polarization into Th2 or Th17 even in the presence of HDMs. Moreover, the *in vivo* results demonstrated the ability of GliSODin® to reduce differentiated Th2 and Th17 cell reactivation by reducing their proliferation, confirming previous studies showing that ROS upregulates costimulatory surface molecules CD40, CD86, and MHCII and promote T-cell proliferation (30) and polarization (31). In addition, we observed that GliSODin®-primed DCs maintained Treg polarization compared with unprimed DCs exposed to HDM. This result could be explained by the ability of GliSODin® to induce the production of IL-10 (14), thus promoting Treg-cell expansion (32). However, we did not measure the ability of GliSODin® to modulate the DC expression of CD40, CD86, MHCII, and IL-10, and further investigations are needed to address this question. The early sensitization phase of allergic asthma is characterized by DC-induced Th2 cells that produce IL-4 and promote B-cell switching toward allergen-specific IgE-secreting cells (33). In our study, we observed that GliSODin® prevented increases in HDM-specific IgE in the serum of HDM-induced asthmatic mice. This lack of allergen-specific IgE after allergen sensitization and challenge may be due to GliSODin®. We previously mentioned that ROS were responsible for DC-induced Th2-cell polarization. Moreover, SOD from GliSODin® is likely to be the enzyme that detoxifies the ROS that induce DC-mediated Th2 polarization, and GliSODin® can be biologically active throughout the mouse model timeline (13). Previous studies using GliSODin® demonstrated its ability to successfully reduce ROS and ROS-related outcomes such as inflammation in animal models and humans (14, 34, 35).

In conclusion, we obtained similar results with wheat GliSODin® (data not shown), and both wheat SOD and melon SOD GliSODin® are preventive agents in allergic asthma. However, this is a pilot study using a small number of mice, and it would be necessary to realize this study on a larger cohort of animals to confirm our results. Nevertheless, our positive results demonstrated that further studies in human are needed to confirm the beneficial effects of GliSODin on allergic asthma.

## Data Availability

The original contributions presented in the study are included in the article/Supplementary Material, further inquiries can be directed to the corresponding author.

## References

[1] Birben E Sahiner UM Sackesen C Erzurum S Kalayci O. Oxidative stress and antioxidant defense. World Allergy Organ J. (2012) 5(1):9–19. 10.1097/WOX.0b013e3182439613 23268465 PMC3488923

[2] Forni C Facchiano F Bartoli M Pieretti S Facchiano A D'Arcangelo D Beneficial role of phytochemicals on oxidative stress and age-related diseases. Biomed Res Int. (2019) 2019:8748253. 10.1155/2019/8748253 31080832 PMC6475554

[3] Finkel T Holbrook NJ. Oxidants, oxidative stress and the biology of ageing. Nature. (2000) 408(6809):239–47. 10.1038/35041687 11089981

[4] Rahman K. Studies on free radicals, antioxidants, and co-factors. Clin Interv Aging. (2007) 2(2):219–36. 18044138 PMC2684512

[5] Pham-Huy LA He H Pham-Huy C. Free radicals, antioxidants in disease and health. Int J Biomed Sci. (2008) 4(2):89–96. 23675073 PMC3614697

[6] Siroux V Boudier A Bousquet J Bresson JL Cracowski JL Ferran J Phenotypic determinants of uncontrolled asthma. J Allergy Clin Immunol. (2009) 124(4):681–7.e3. 10.1016/j.jaci.2009.06.010 19665764

[7] Lang DM. Severe asthma: epidemiology, burden of illness, and heterogeneity. Allergy Asthma Proc. (2015) 36(6):418–24. 10.2500/aap.2015.36.3908 26534747

[8] Ammar M Bahloul N Amri O Omri R Ghozzi H Kammoun S Oxidative stress in patients with asthma and its relation to uncontrolled asthma. J Clin Lab Anal. (2022) 36(5):e24345. 10.1002/jcla.24345 35318723 PMC9102642

[9] Schieber M Chandel NS. ROS function in redox signaling and oxidative stress. Curr Biol. (2014) 24(10):R453–62. 10.1016/j.cub.2014.03.034 24845678 PMC4055301

[10] Bazan-Socha S Wojcik K Olchawa M Sarna T Pieta J Jakiela B Increased oxidative stress in asthma-relation to inflammatory blood and lung biomarkers and airway remodeling indices. Biomedicines. (2022) 10(7):1499–515. 10.3390/biomedicines10071499 35884804 PMC9312921

[11] Yasui K Kobayashi N Yamazaki T Agematsu K Matsuzaki S Ito S Superoxide dismutase (SOD) as a potential inhibitory mediator of inflammation via neutrophil apoptosis. Free Radic Res. (2005) 39(7):755–62. 10.1080/10715760500104066 16036355

[12] Romao S. Therapeutic value of oral supplementation with melon superoxide dismutase and wheat gliadin combination. Nutrition. (2015) 31(3):430–6. 10.1016/j.nut.2014.10.006 25701330

[13] Vouldoukis I Conti M Krauss P Kamate C Blazquez S Tefit M Supplementation with gliadin-combined plant superoxide dismutase extract promotes antioxidant defences and protects against oxidative stress. Phytother Res. (2004) 18(12):957–62. 10.1002/ptr.1542 15742357

[14] Vouldoukis I Lacan D Kamate C Coste P Calenda A Mazier D Antioxidant and anti-inflammatory properties of a *Cucumis melo* LC. extract rich in superoxide dismutase activity. J Ethnopharmacol. (2004) 94(1):67–75. 10.1016/j.jep.2004.04.023 15261965

[15] Seo YS Kim HS Lee AY Chun JM Kim SB Moon BC *Codonopsis lanceolata* attenuates allergic lung inflammation by inhibiting Th2 cell activation and augmenting mitochondrial ROS dismutase (SOD2) expression. Sci Rep. (2019) 9(1):2312. 10.1038/s41598-019-38782-6 30783201 PMC6381190

[16] Jarikre TA Ohore GO Oyagbemi AA Emikpe BO. Evaluation of oxidative stress in caprine bronchoalveolar lavage fluid of pneumonic and normal lungs. Int J Vet Sci Med. (2017) 5(2):143–7. 10.1016/j.ijvsm.2017.09.001 30255063 PMC6137846

[17] Tiwari M Dwivedi UN Kakkar P. *Tinospora cordifolia* extract modulates COX-2, iNOS, ICAM-1, pro-inflammatory cytokines and redox status in murine model of asthma. J Ethnopharmacol. (2014) 153(2):326–37. 10.1016/j.jep.2014.01.031 24556222

[18] Wang K Wang L Zhao G Liu Y Wang F Song H Mechanistic study of salidroside on ovalbumin-induced asthmatic model mice based on untargeted metabolomics analysis. Food Funct. (2023) 14(1):413–26. 10.1039/D2FO02225G 36515134

[19] Xu C Song Y Wang Z Jiang J Piao Y Li L Pterostilbene suppresses oxidative stress and allergic airway inflammation through AMPK/Sirt1 and Nrf2/HO-1 pathways. Immun Inflamm Dis. (2021) 9(4):1406–17. 10.1002/iid3.490 34342160 PMC8589405

[20] Park HS Kim SR Lee YC. Impact of oxidative stress on lung diseases. Respirology. (2009) 14(1):27–38. 10.1111/j.1440-1843.2008.01447.x 19144046

[21] Gonzalo JA Lloyd CM Wen D Albar JP Wells TN Proudfoot A The coordinated action of CC chemokines in the lung orchestrates allergic inflammation and airway hyperresponsiveness. J Exp Med. (1998) 188(1):157–67. 10.1084/jem.188.1.157 9653092 PMC2525544

[22] Henderson WR Chi EY Teo JL Nguyen C Kahn M. A small molecule inhibitor of redox-regulated NF-*κ*B and activator protein-1 transcription blocks allergic airway inflammation in a mouse asthma model. J Immunol. (2002) 169(9):5294–9. 10.4049/jimmunol.169.9.5294 12391249

[23] Wang JC Zhao Y Chen SJ Long J Jia QQ Zhai JD AOPPs induce MCP-1 expression by increasing ROS-mediated activation of the NF-*κ*B pathway in rat mesangial cells: inhibition by sesquiterpene lactones. Cell Physiol Biochem. (2013) 32(6):1867–77. 10.1159/000356619 24356300

[24] Loukides S Bouros D Papatheodorou G Panagou P Siafakas NM. The relationships among hydrogen peroxide in expired breath condensate, airway inflammation, and asthma severity. Chest. (2002) 121(2):338–46. 10.1378/chest.121.2.338 11834641

[25] Matsuo K Kitahata K Kaibori Y Arima Y Iwama A Ito M CCR4 involvement in the expansion of T helper type 17 cells in a mouse model of psoriasis. J Invest Dermatol. (2021) 141(8):1985–94. 10.1016/j.jid.2020.12.034 33662381

[26] Vestergaard C Bang K Gesser B Yoneyama H Matsushima K Larsen CG. A Th2 chemokine, TARC, produced by keratinocytes may recruit CLA+CCR4+lymphocytes into lesional atopic dermatitis skin. J Invest Dermatol. (2000) 115(4):640–6. 10.1046/j.1523-1747.2000.00115.x 10998136

[27] Qi XF Teng YC Yoon YS Kim DH Cai DQ Lee KJ. Reactive oxygen species are involved in the IFN-gamma-stimulated production of Th2 chemokines in HaCaT keratinocytes. J Cell Physiol. (2011) 226(1):58–65. 10.1002/jcp.22303 20625996

[28] Kapsenberg ML. Dendritic-cell control of pathogen-driven T-cell polarization. Nat Rev Immunol. (2003) 3(12):984–93. 10.1038/nri1246 14647480

[29] van Beelen AJ Zelinkova Z Taanman-Kueter EW Muller FJ Hommes DW Zaat SA Stimulation of the intracellular bacterial sensor NOD2 programs dendritic cells to promote interleukin-17 production in human memory T cells. Immunity. (2007) 27(4):660–9. 10.1016/j.immuni.2007.08.013 17919942

[30] Rutault K Alderman C Chain BM Katz DR. Reactive oxygen species activate human peripheral blood dendritic cells. Free Radic Biol Med. (1999) 26(1–2):232–8. 10.1016/S0891-5849(98)00194-4 9890657

[31] Tang H Cao W Kasturi SP Ravindran R Nakaya HI Kundu K The T helper type 2 response to cysteine proteases requires dendritic cell-basophil cooperation via ROS-mediated signaling. Nat Immunol. (2010) 11(7):608–17. 10.1038/ni.1883 20495560 PMC3145206

[32] Hsu P Santner-Nanan B Hu M Skarratt K Lee CH Stormon M IL-10 potentiates differentiation of human induced regulatory T cells via STAT3 and Foxo1. J Immunol. (2015) 195(8):3665–74. 10.4049/jimmunol.1402898 26363058

[33] Del Prete G Maggi E Parronchi P Chretien I Tiri A Macchia D IL-4 is an essential factor for the IgE synthesis induced in vitro by human T cell clones and their supernatants. J Immunol. (1988) 140(12):4193–8. 10.4049/jimmunol.140.12.4193 2967330

[34] Nakamura A Kitamura N Yokoyama Y Uchida S Kumadaki K Tsubota K Melon GliSODin® prevents diet-induced NASH onset by reducing fat synthesis and improving liver function. Nutrients. (2019) 11(8):1779–90. 10.3390/nu11081779 31374969 PMC6722950

[35] Fontas E Montaudie H Passeron T. Oral gliadin-protected superoxide dismutase in addition to phototherapy for treating non-segmental vitiligo: a 24-week prospective randomized placebo-controlled study. J Eur Acad Dermatol Venereol. (2021) 35(8):1725–9. 10.1111/jdv.17331 33931900 PMC8360035

