# Supplementary material for: GliSODin® prevents airway inflammation by inhibiting T-cell differentiation and activation in a mouse model of asthma
Source: Front Allergy. 2023 Jun 6;4:1199355. doi: 10.3389/falgy.2023.1199355 (PMC10279865; doi:10.3389/falgy.2023.1199355)

**Supplementary Material**

**Supplementary data S1.** GliSODin® affects the humoral response by modulating specific IgA and IgG2a levels but not IgG1 levels. Specific IGA (A), IgG1 (B) and IgG2a (C) levels in mouse serum were quantified by ELISA. The white bar indicates the control (CTL), the light grey bar indicates the control plus GliSODin® treatment (CTL+Gli), the black bar indicates HDM sensitized mice (HDM), and the grey bar indicates HDM sensitized mice with GliSODin® treatment (HDM+Gli). The data are represented as the mean ± SEM, n=6 mice per group for all groups. *p<0.05, **p<0.01, ***p<0.005, ****p<0.001.


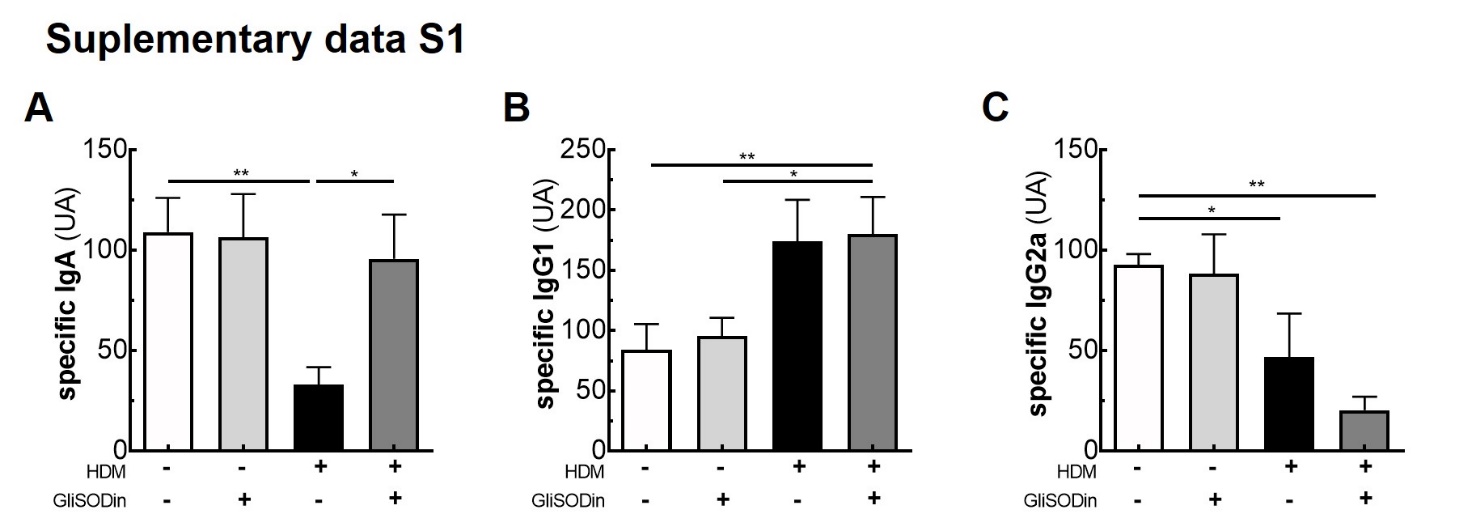


**Supplementary data S2.** GliSODin® does not have an effect on T CD8+ lymphocytes. CD8+ IFNγ+ lymphocytes in the lung were quantified by flow cytometry (A). The white bar indicates the control (CTL), the light grey bar indicates the control plus GliSODin® treatment (CTL+Gli), the black bar indicates HDM sensitized mice (HDM), and the grey bar indicates HDM sensitized mice with GliSODin® treatment (HDM+Gli). The data are represented as the mean ± SEM, n=8 mice per groups for all groups.


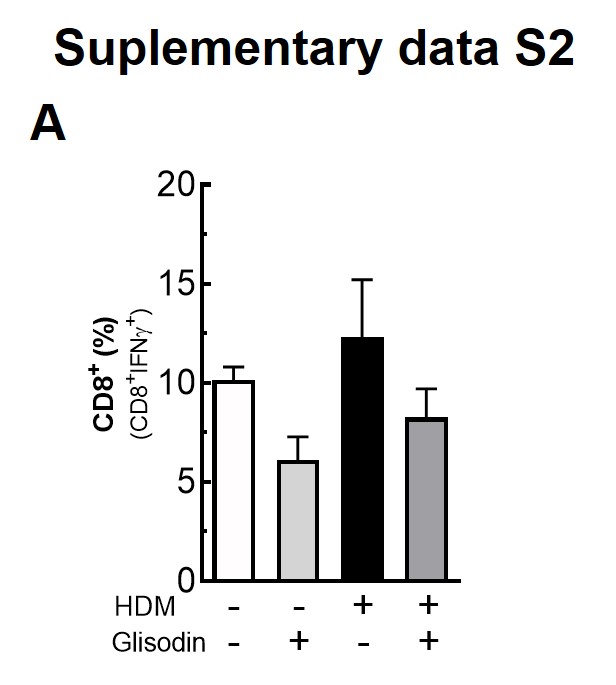


**Supplementary data S3.** Gating strategy of Th1, Th2, Th17 and Treg cells analysis


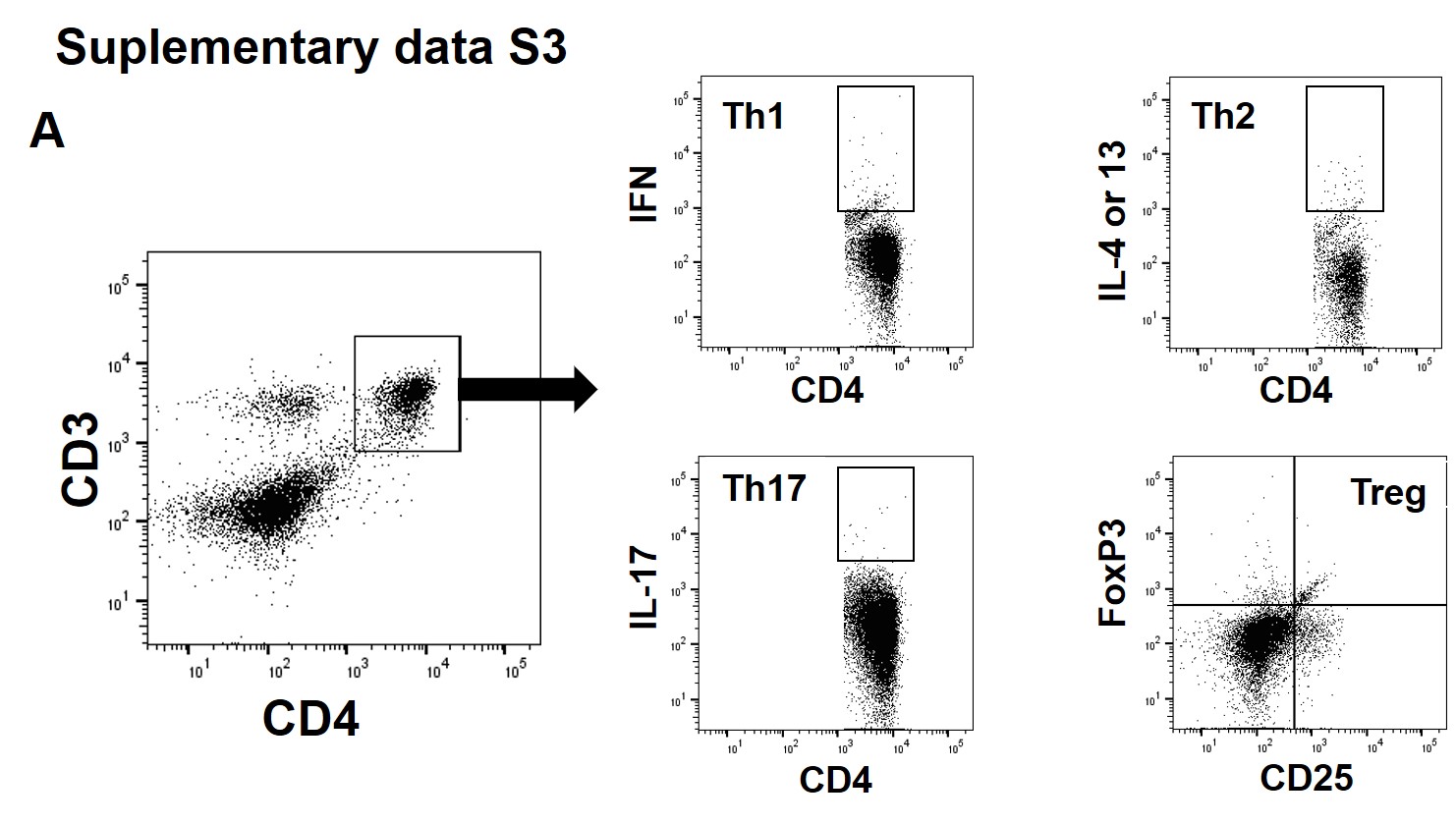

Supplement: Supplementary file 1 [file Datasheet1.docx]
